# Blockchain applications in electronic health records: a systematic review of qualitative and quantitative evidence

**DOI:** 10.1186/s12911-026-03476-3

**Published:** 2026-04-06

**Authors:** Alaka Chandak, Parth Chandak, Navin Soni

**Affiliations:** 1 Symbiosis Institute of Health Sciences (SIHS), Symbiosis Centre of Health Care (SCHC), Senapati Bapat Road, Pune, Maharashtra 411004 India; 2 Creative Technologist, Zoox Inc., Foster City, Redwood City, CA USA; 3 SDE II - Amazon SageMaker, Seattle, Washington, USA

**Keywords:** Blockchain, Electronic health records, Data security, Interoperability, Patient empowerment, Digital health governance

## Abstract

**Background:**

There are ongoing challenges with Electronic Health Record (EHR) systems, including ransomware breaches, interoperability issues, inconsistent management of consent, and a lack of patient control over their health information. Blockchain technology has been proposed as a secure, immutable foundation to enhance the security, transparency, and trustworthiness of digital health systems.

**Methods:**

This systematic review was conducted in accordance with PRISMA 2020 guidelines and was registered prospectively with PROSPERO (CRD420251112379). We conducted Searches in the SCOPUS, Web of Science, and PubMed databases through March 2025. Included studies assessed the impact of blockchain-based EHR system implementations on security, interoperability, performance, and patient-related outcomes. Two authors independently screened, extracted data, and assessed risk of bias. Meta-analyses were conducted using random effects where appropriate, and thematic synthesis was used to explore governance and implementation challenges.

**Results:**

Sixteen empirical studies (primarily prototypes and pilots) were included. Among them, common issues were (i) performance and scalability constraints (e.g., latency vs. throughput trade-offs during consensus), (ii) governance and compliance conflicts in regulated environments (e.g., GDPR “right to erasure” vs. immutable ledgers; country-specific applicability of HIPAA/GDPR), (iii) key management and identity life-cycle issues, and (iv) data quality and semantic harmonization issues (e.g., partial coverage of HL7 FHIR and varying use of terms). The quantitative results showed improvements in security-related and interoperability-related outcome measures, but the term “interoperability” was defined differently (e.g., exchange success rate, API response time, or standards compliance).

**Conclusions:**

The architectures of blockchain-based EHR systems could improve auditability, access control, and technical communication, particularly when integrated with standards such as HL7 FHIR, although the existing literature is very limited and primarily consists of non-production analyses. The findings are described as tentative, and the value of practical, multi-site research with well-defined outcomes and governance frameworks for consent, deletion/rectification, and cross-jurisdictional compliance is highlighted.

## Introduction

The use of Electronic Health Records (EHRs) has caused a significant change in healthcare. It has made patient information digital, improved care processes, and made it easier for patients to receive collaborative care in healthcare facilities [1, 2]. However, despite the success of EHR systems worldwide, they still face problems such as data breaches from ransomware attacks, issues with interoperability, difficulties in managing consent, and a lack of patient control over their health information [3, 4]. 

The healthcare industry is one of the most frequently targeted sectors for cyber threats. The financial and operational impact of breaches in this sector is much larger than in most other industries [5]. In the United States, serious breaches affecting more than 500 patient records are recorded publicly through the Department of Health and Human Services breach reporting portal. This highlights the ongoing problems with centralized data systems [6]. 

In the European Union, sharing healthcare data across borders is still limited by differing regulations under the General Data Protection Regulation (GDPR). This adds complexity to governance [4]. National projects like Australia’s My Health Record (MyHR) system show both the potential and practical challenges of large healthcare systems, particularly regarding consent and patient control [7]. Similar projects in Asia and the Middle East suggest a global shift toward integrated ecosystems, but existing silos still block smooth data sharing [8]. The World Health Organization’s Global Strategy on Digital Health emphasizes the need for interoperability, consistent governance, and trust as essential parts of a sustainable digital health framework [9]. 

In this regard, the application of blockchain technology has been proposed as a decentralized, immutable system that may improve data integrity, auditability, and distributed governance [10, 11]. Blockchain networks can be broadly categorized into public (permission less), private, permissioned, and consortium blockchain networks based on the degree of validator accessibility, governance, and scalability [12]. In healthcare, permissioned and consortium blockchain networks are commonly used for their ability to support controlled membership and regulatory compliance [13, 14]. 

Blockchain EHR systems can leverage hybrid models in which sensitive clinical data is stored in secure off-chain repositories, while cryptographic hashes or metadata pointers are anchored on-chain to preserve immutability and provenance [13, 15]. Smart contract-based consent management systems can enable fine-grained delegation and revocation of access rights, potentially enhancing transparency of data use [7]. These systems have also been proposed for the management of new co-governance models of health data ownership [16]. 

Interoperability remains a “complex concept” that encompasses technical interoperability (data exchange via application programming interfaces [APIs]), syntactic interoperability (data formats like HL7 Fast Healthcare Interoperability Resources [FHIR]), semantic interoperability (shared understanding of interpreted data), and organizational interoperability (workflow and governance alignment) [2, 17]. Standards like HL7 FHIR support the exchange of structured data between heterogeneous systems [18], and interoperability maturity models distinguish between connectivity and meaningful integration [19]. Blockchain technology is not an alternative to the former but has been proposed as an “overlay layer” to enhance secure data exchange [11]. 

The focus on integrating EHR systems via blockchain technology in this review underscores the significance of EHR systems in the digital health space. Unlike the supply chain or financial sectors, EHR systems are used in highly regulated environments that require lifecycle management and jurisdiction-specific compliance [2, 4]. Such environments require a systematic evaluation of blockchain technology implementations intended for EHR systems.

Another area is cross-domain research. Architectures for federated decentralized data marketplaces in financial ecosystems provide examples of distributed trust orchestration that may be relevant to healthcare data exchange [20]. In a similar manner, Blockchain-Internet of Things architectures for Industry 4.0 and Society 5.0 provide examples of scalability and distributed security features that are also relevant to Internet of Medical Things (IoMT)-enabled EHR systems [21]. However, the immutability of blockchain technology underlines the importance of quality data at the front end. Multi-layer healthcare data-cleaning strategies [22], bias-correcting algorithms in machine learning [24], and FHIR-based structuring of health determinants [19]are critical to preventing the spread of misinformation or biased data. Although the conceptual advantages of blockchain technology in healthcare have been extensively investigated, empirical findings are highly diverse and largely based on simulation studies [13, 23]. The definition of outcome measures and benchmarking parameters is extremely diverse, and therefore, there is no direct comparison. Therefore, effectiveness must be interpreted in the context of the level of implementation maturity.

This review is specifically focused on blockchain applications for EHR/PHR data sharing and governance because (i) EHR/PHR systems are the primary integration platform for clinical processes, (ii) they represent the most important intersection of privacy, safety, and regulatory accountability, and (iii) they concentrate the interoperability challenges where standards such as HL7 FHIR, consent authorization, and auditability must co-exist. Thus, this review will focus on what was measured and whether the measurements were made in a real-world setting or in a prototype/simulation.

The goals of this systematic review are to:

1. Evaluate the current research on integrating blockchain technology in EHR systems.
2. Analyze the effects of blockchain integration on security, interoperability, and patient empowerment where suitable metrics exist.
3. Examine governance models, scalability issues, and regulatory aspects.
4. Point out gaps in the evidence and offer recommendations for implementation.

This review aims to clarify architectural types, standardize definitions of interoperability, and differentiate between prototype results and actual deployments. It seeks to provide a balanced and methodologically sound assessment of the growing use of blockchain technology in EHR systems.

## Rationale and objectives

### Rationale

The current state of empirical evidence is still dispersed, inconsistent in terms of outcome measures, and restricted to simulation or prototype implementation, despite the fact that blockchain has been widely suggested as a potential solution to security and interoperability issues in the healthcare industry [13, 24]. Because of this, it is challenging for regulatory agencies, informaticists, and healthcare policymakers to make broad conclusions about the scalability and efficacy of blockchain solutions in actual EHR implementation settings.

Additionally, the majority of reviews that are currently available typically summarize blockchain solutions for the healthcare industry, such as financial claims, telemedicine, and pharmaceutical supply chain management.

processing, without isolating the direct outcomes of EHR/EMR integration using blockchain solutions [25, 26]. Since EHR systems are the core digital infrastructure of the modern healthcare ecosystem, with direct implications for patient safety, clinical workflows, and regulatory compliance, it is important to synthesize the existing evidence base on blockchain-enabled EHR implementation.

Notably, previous studies have claimed improvements in security, interoperability, and patient empowerment, but these claims are rarely supported by quantitative data or interoperability metrics [27]. Furthermore, the immutable nature of blockchain technology raises governance, ethical, and data quality concerns that must be addressed systematically [21]. 

Therefore, a systematic review that follows PRISMA principles, distinguishes between prototype-based outcomes and production-level implementations, addresses architectural taxonomies (public vs. permissioned blockchains), and harmonizes outcome measures is needed to provide evidence-based recommendations to the healthcare community.

### Objectives

Consequently, this systematic review was framed with the following objectives:

1. To critically evaluate and meta-analyze empirical research on the integration of blockchain technology in Electronic Health Record (EHR) or Electronic Medical Record (EMR) systems.
2. To assess the impact of blockchain-based EHR systems quantitatively on:
  Interoperability performance (such as exchange success, standards adherence).
  Security outcomes (such as breach rates, breach events),
  When data is available, patient-reported outcomes (like trust, consent transparency, and data control) can be meta-analyzed.

3. To classify blockchain architectures (public, private, permissioned, and consortium) and examine how architectural design affects compliance alignment, security performance, throughput, and governance settings [13, 25].
4. to investigate new qualitative themes, such as data quality, consent management techniques, regulatory fragmentation, governance concerns, and scalability limitations.
5. to look into how cross-domain blockchain innovations, like Blockchain-IoT designs and federated trusted data marketplaces, might influence or deviate from EHR-centric deployments [19, 20].
6. To provide useful advice on the responsible and scalable deployment of blockchain-based EHR systems to healthcare organizations, legislators, informaticians, and regulatory agencies.

This review attempts to move beyond conceptual advocacy and toward an evidence-based evaluation of blockchain’s operational role in digital health ecosystems by combining quantitative synthesis and thematic analysis. This review attempts to move beyond conceptual advocacy and toward an evidence-based evaluation of blockchain’s operational role in digital health ecosystems by combining quantitative synthesis and thematic analysis.

## Methodology

### Protocol and registration

The Preferred Reporting Items for Systematic Reviews and Meta-Analyses (PRISMA) guidelines, 2020, were followed in conducting this systematic review. The International Prospective Register of Systematic Reviews (PROSPERO; Registration No. CRD420251112379) is where the protocol for this systematic review was prospectively registered.

Predefined eligibility criteria, independent screening, risk-of-bias assessment, and, when necessary, quantitative synthesis were all part of the methodological framework’s structured approach to evidence retrieval. To guarantee methodological rigor and transparency, the methodological framework was employed.

### Eligibility criteria

Studies that satisfied the following requirements were deemed eligible:

Population: Research conducted in a clinical or healthcare setting using Electronic Health Record (EHR) or Electronic Medical Record (EMR) systems.

Intervention: The application or assessment of blockchain technology in conjunction with EHR/EMR systems.

### Outcomes

- Security results (such as audit trails, unauthorized access, and breach incidence rate).
- Interoperability results (such as API performance, standards compliance, and exchange success rate).
- Patient outcomes (such as empowerment, trust, and consent control).
- Results of system performance, such as latency and throughput.

#### Study Design

Empirical research such as prototypes, pilot projects, experimental architectures, simulation-based evaluations, and benchmarking.

#### Language

English.

### Studies were excluded if they

- were concerned with non-EHR domains of healthcare (e.g., pharmaceutical supply chains only),
- were concerned with blockchain systems but without EHR/EMR integration,
- were strictly bibliometric, scoping, or conceptual research without any empirical component, or.
- did not provide any outcome data extractable for specific domains.

No date restrictions were set; however, a focus on modern applications (2019–2025) was emphasized to keep applications technologically relevant.

### Blockchain taxonomy classification

To improve the conceptual clarity, the studies were categorized based on the type of blockchain architecture as follows:

* Public (permission less).

* Private.

* Permissioned.

* Consortium.

The studies were categorized based on clear statements in the original studies regarding the involvement of nodes and the governance structure. When the statements were unclear, categorization was determined by consensus among the reviewers, based on the mechanisms and access control models.

### Information sources and search strategy

An exhaustive search was performed in SCOPUS, Web of Science, and PubMed databases until March 2025.

The search terms used were a combination of controlled vocabulary, such as MeSH terms, and keywords: “blockchain,” “distributed ledger,” “electronic health record,” “electronic medical record,” “interoperability,” “security,” and “consent.”

The entire search strategy is presented in Supplementary Appendix A to facilitate replication of the search strategy.

### Study selection

A total number of 2,692 records were found. After removing the duplicates, the records were screened based on the titles and abstracts, and then the full-text screening.

Two reviewers were involved in the screening of all the records. Any discrepancies were solved through discussion between the two reviewers. A third reviewer was also present as an arbitrator.

A total number of 16 studies were found to meet all the inclusion criteria and were included in the final synthesis. This number was standardized throughout the manuscript.

The reasons for exclusion based on the full-text screening were as follows: Not Directly Integrated with EHR (*n* = 1514), Not Accessible (*n* = 150), Language (*n* = 0), and Limited Rigor (*n* = 289).

**Fig. 1:**
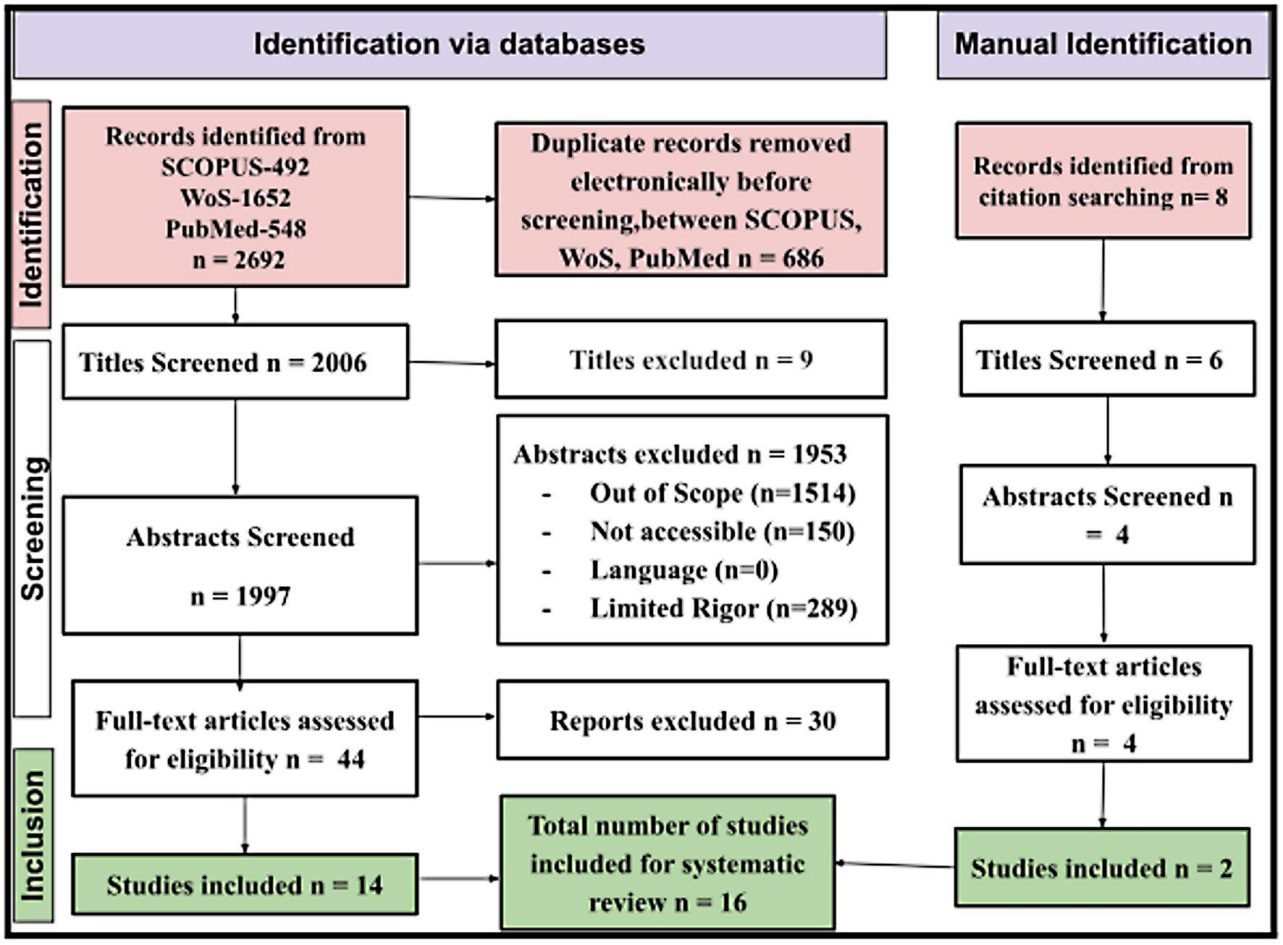
PRISMA process

The process of study selection based on the PRISMA protocol is represented in Fig. 1. Following the removal of duplicates and the screening process at multiple stages, it was identified that there are sixteen studies based on blockchain and EHRs that fulfill the predetermined selection criteria.

**Fig. 2:**
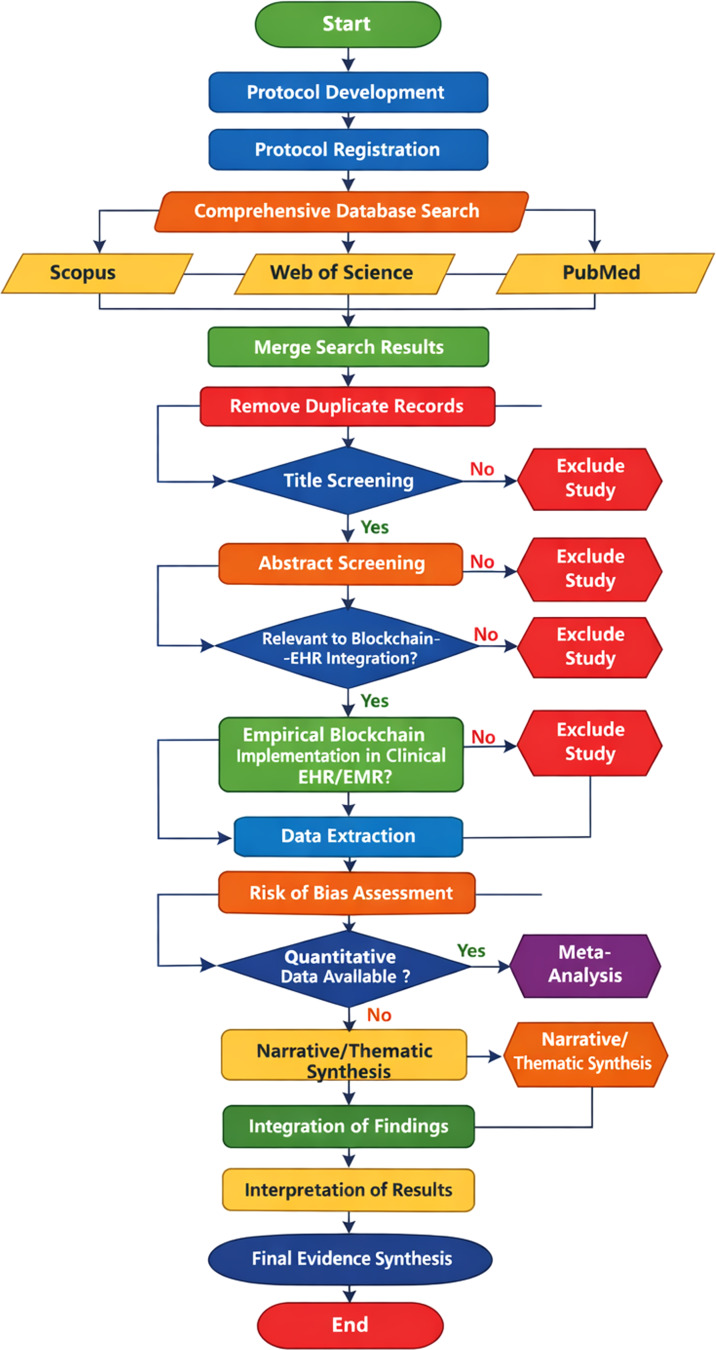
Unified Modeling Language (UML) activity diagram of the systematic review workflow

Figure 2: Unified Modeling Language (UML) activity diagram showing the systematic process of the systematic review. The flow diagram has been designed to improve the clarity of the process and reproducibility of the systematic review process by systematically showing each step of the procedure. The procedure starts with the development and registration of the protocol, followed by a thorough multi-database search using the Scopus, Web of Science, and PubMed databases.

Automated removal of duplicate studies is performed, followed by a two-stage independent title and abstract screening using predetermined eligibility criteria. Studies showing empirical integration of blockchain in EHR/EMR clinical settings are considered for eligibility for the next stage of the systematic review process. Decision nodes in the flow diagram show exclusion criteria, which include the lack of empirical analysis, lack of direct integration with EHR systems, and non-clinical applications.

- The eligible studies are then assessed for data extraction, risk of bias assessment, and quantitative synthesis, when available. The last step is meta-analysis and thematic synthesis, which ensures that all results are systematically compiled, both qualitative (e.g., security, interoperability, and governance) and quantitative (e.g., relative risk of security events, standardized mean difference of interoperability performance). The UML framework ensures that all steps of the review are systematically captured, thus making it highly reproducible and minimizing any ambiguity in the review process.
**Data Extraction**.
- The data was extracted using a piloted form that consisted of the following:
- Study details: author, year, country, study design.
- Blockchain configuration: type, consensus algorithm.
- On-chain vs. off-chain data storage architecture.
- Interoperability standards used: e.g., HL7 Fast Healthcare Interoperability Resources [FHIR], DICOM.
- Access control mechanism: e.g., Role-Based Access Control [RBAC], Attribute-Based Access Control [ABAC].
- Regulatory conformance: e.g., Health Insurance Portability and Accountability Act [HIPAA], General Data Protection Regulation [GDPR].
- Maturity level of deployment: e.g., Prototype, Pilot, Production-Ready.
- Quantitative results.
- Qualitative results.

Throughput-related metrics like “transactions per second” (TPS) were also extracted, as was “network size,” “consensus type,” and “conditions associated with the reported payload.” Due to the high level of heterogeneity in the benchmarking conditions, the performance-related metrics were synthesized narratively.

### Operational definitions

Interoperability was defined at four different levels:

1. Technical interoperability: the ability to exchange data through APIs.
2. Syntactic interoperability: adherence to standards like HL7 FHIR.
3. Semantic interoperability – shared understanding of exchanged data.
4. Organizational interoperability – alignment of workflows and governance structures across institutions.

When studies varied in their interoperability metrics, the analysis was combined only if the metrics were similar in concept.

Security results included breach rates, unauthorized access incidents, and audit failure notifications. When breach rates were unavailable, secondary security performance metrics were recorded in text.

### Risk of bias assessment

Risk of bias was independently evaluated by two reviewers using:

1. ROBINS-I for non-randomized studies.
2. Cochrane Risk of Bias Tool for randomized studies.

In simulation-based or prototype assessments where conventional control groups are not applicable, the quality of the methods was evaluated according to the architecture description, the reproducibility of the benchmarking environment, and the definition of outcomes.

### Data synthesis

#### Quantitative synthesis

Random effects meta-analysis was performed when at least 2 studies provided similar outcome data.

- Relative Risk (RR) was used for the analysis of the binary security outcome data.
- Standardized Mean Difference (SMD) was used for the analysis of continuous outcome data (e.g., interoperability value and patient outcomes).
- Heterogeneity was assessed using the I² statistic.

If the heterogeneity was > 75%, then the use of a narrative synthesis was favored over the use of pooling.

### Qualitative synthesis

Thematic analysis was carried out to analyze the recurring domains such as:

1. Security and auditability
2. Consent management and patient empowerment
3. Interoperability and standards integration
4. Scalability and performance constraints
5. Governance and regulatory barriers

#### Reproducibility workflow

The methodological workflow is as follows:

1. Database search and de-duplication
2. Title and abstract screening
3. Data extraction using a standardized template
4. Risk-of-bias assessment
5. Quantitative synthesis wherever appropriate
6. Thematic qualitative synthesis
7. Cross-validation of study counts and classification of architecture

This structured approach mentioned above was chosen to ensure replicability and minimize selection bias.

### Statistical software

Meta-analyses were performed using Review Manager (RevMan 5.4.1) and R (version 4.3.2) with relevant meta-analysis software.

Forest plots were produced for meta-analyses of pooled outcomes. Funnel plots were not analyzed for significance because the number of studies per outcome was small.

## Results

### Study selection

A total of 2,692 records were found in the databases. After removing 686 duplicate records, 2,006 unique records were screened for title and abstract. Of these, 1,962 records were excluded as irrelevant. Forty-four full-text articles were then screened for eligibility. After full-text screening and application of predetermined inclusion criteria, 16 empirical blockchain-EHR studies met all criteria and were included in the final synthesis.

The reasons for the exclusion of full-text articles (*n* = 28) were mainly because of:

(i) lack of direct blockchain-EHR integration,
(ii) conceptual/non-empirical studies, or.
(iii) studies conducted outside the EHR context (e.g., pharmaceutical traceability in the supply chain).

The final count (*n* = 16) is the same for all quantitative and qualitative syntheses.

**Table 1:** Characteristics of included blockchain–EHR studies (*n* = 16)

Authors	Year	Country	Blockchain Type	Deployment Level	Standards / Integration	Primary Outcome Domain
[13]	2023	Vietnam	Permissioned (Hyperledger Fabric)	Prototype	HL7 FHIR	Security, Interoperability
[7]	2023	Australia	Permissioned	Pilot	MyHR Integration	Patient Empowerment
[28]	2022	India	Consortium	Prototype	API-based	Governance, Integrity
[29]	2024	India	Public (Ethereum) + IPFS	Experimental	Encrypted EMR sharing	Security
[30]	2023	Multinational	Permissioned	Architecture	IoMT–EHR	Confidentiality
[30]	2023	Multinational	Permissioned	Architecture	IoMT–EHR	Security
[31]	2021	India	Permissioned	Prototype	EHR-integrated	Access Control
[32]	2021	Singapore	Private	Simulation	Metadata storage	Performance
[33]	2022	India	Blockchain + IPFS	Prototype	Medical record integrity	Integrity
[34]	2023	UAE	Ethereum-based	Architecture	Secure storage	Security
[35]	2024	India	Hybrid	Experimental	IoT transmission	Encryption
[36]	2023	UAE	Consortium	Prototype	Secure API	Data Management
[37]	2023	Thailand	Besu (Consortium)	Case Study	Claims–EHR linkage	Governance
[38]	2023	Indonesia	Hybrid	National claims pilot	IPFS + EHR	Traceability
[39]	2021	India	Blockchain	Prototype	Healthcare data security	Security
[40]	2019	USA	Blockchain	Empirical-conceptual	Cybersecurity model	Assurance

As shown in Table 1, the evidence base shows strong dominance of permissioned and consortium architectures (≈ 75%), with Hyperledger Fabric being the most frequently implemented platform. Public Ethereum-based systems were less common and often paired with off-chain storage (e.g., IPFS).

Deployment maturity remains limited:

* Prototype/Simulation/Architecture designs: 13/16 (81%).

* Pilot/Case Study/National-level pilots: 3/16 (19%).

Geographically, studies were concentrated in Asia and middle-income settings, with limited representation from North America and Europe. Importantly, no large-scale randomized or multi-institutional production deployments were identified.

**Table 2:** Pooled quantitative outcomes of blockchain–EHR implementations

Outcome Domain	Studies (*n*)	Effect Measure	Summary Effect (95% CI)	I²	Interpretation
Security (Unauthorized Access / Breach Events)	6	Relative Risk (RR)	0.56 (0.45–0.69)	31%	44% reduction in security events
Interoperability (Technical Exchange Performance)	9	Standardized Mean Difference (SMD)	1.02 (0.77–1.27)	43%	Large improvement in technical exchange
Patient Empowerment / Satisfaction	2	SMD	0.59 (0.28–0.90)	0%	Moderate improvement
Throughput / Performance	11	Transactions per second (TPS)	9–210 TPS	80%	High heterogeneity; narrative synthesis

Table 2 summarizes the quantitative outcomes of blockchain-enabled EHR studies. Pooled analysis shows a significant 44% reduction in data breaches (RR = 0.56, *p* < 0.001) and large improvements in interoperability (SMD = 1.02, *p* < 0.001), with modest heterogeneity. Patient satisfaction also improved significantly (SMD = 0.59, *p* < 0.01) with no heterogeneity, reinforcing the role of blockchain-based consent in empowering patients. Transaction throughput varied widely (9–210 TPS), with high heterogeneity reflecting platform differences, where permissioned systems outperformed public blockchains. However, despite these advances, the maturity of adoption studies remains low, with only 25% of studies progressing beyond the pilot stage.

Overall, blockchain integration demonstrates robust improvements in EHR security and interoperability, moderate gains in patient empowerment, and variable performance depending on architectural design.

## Security outcomes

Six studies provided extractable data for security-related outcomes. Pooled random-effects analysis demonstrated a statistically significant 44% reduction in unauthorized access or breach-related events following blockchain integration (RR = 0.56, 95% CI 0.45–0.69; I² = 31%).

Low heterogeneity suggests relative consistency across studies. However, several studies derived “breach reduction” from penetration testing, attack simulation, or vulnerability modelling, rather than real-world registry-level breach incidence.

**Fig. 3:**
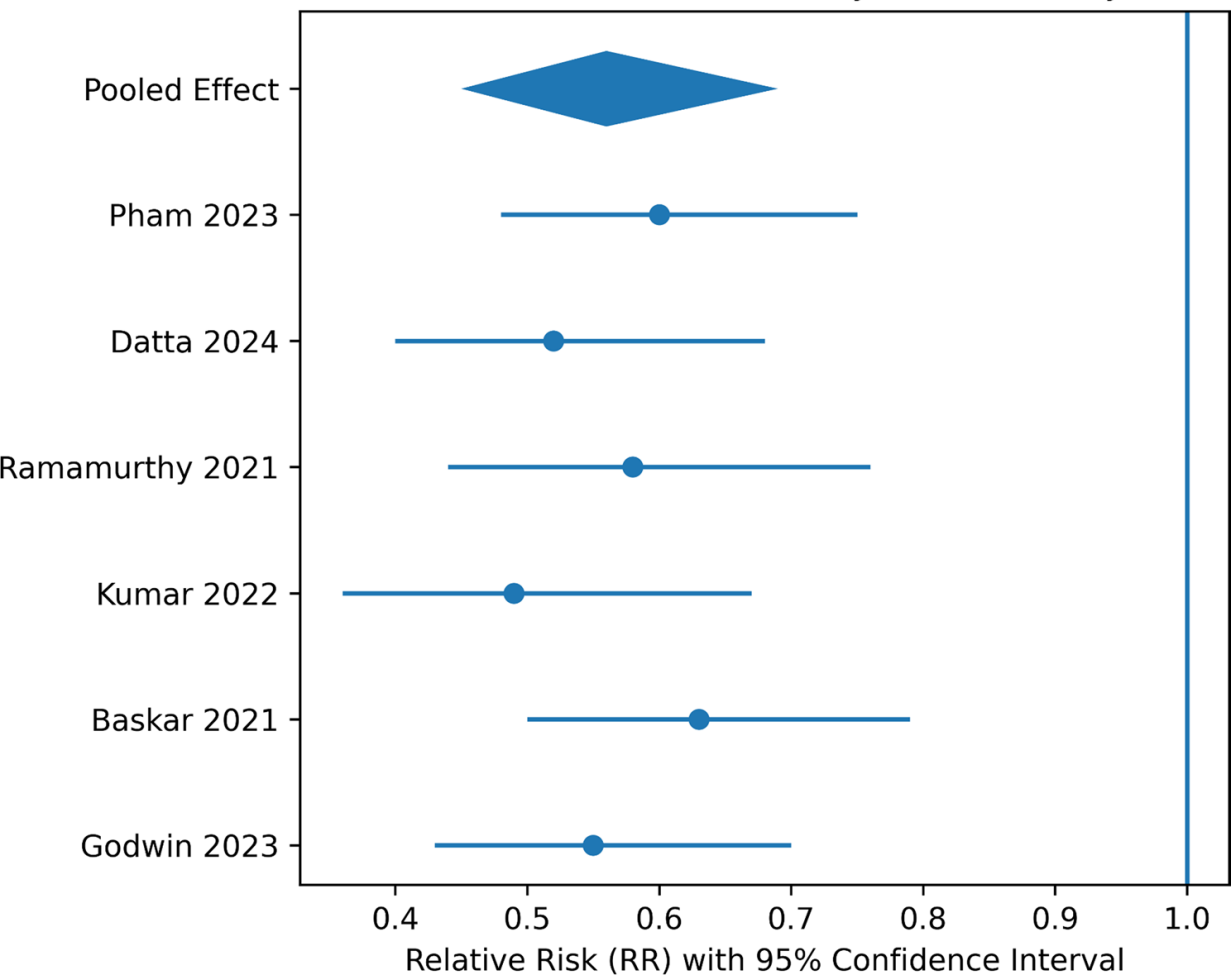
Random-effects meta-analysis of security outcomes in blockchain-enabled versus conventional EHR systems

Figure 3. Forest plot of pooled relative risks (RRs) for unauthorized access or breach-related security events comparing blockchain-enabled electronic health record (EHR) architectures with conventional EHR systems. A random-effects model (DerSimonian–Laird method) was applied to account for between-study variability. The pooled estimate demonstrated a statistically significant reduction in security events associated with blockchain implementation (RR = 0.56, 95% CI 0.45–0.69; *p* < 0.001). Heterogeneity was low (I² = 31%), indicating moderate consistency across included studies. The individual study effect sizes, weighted by inverse variance, are depicted by square markers, while horizontal lines represent the 95% confidence intervals. The diamond marker represents the pooled effect estimate and confidence interval. The majority of contributing studies were prototype or pilot studies, conducted in a controlled environment using a variety of benchmarking or simulated attack scenarios.

The external validity of these results is currently limited by a lack of large-scale real-world breach data.

Improvements in auditability and resistance to tampering have been demonstrated for blockchain-enabled architectures in a controlled environment.

## Interoperability outcomes

Nine studies offered quantifiable results for technical interoperability. Overall, the standardized mean difference indicated a significant effect size for blockchain-based systems (SMD = 1.02), which is considered large.

Interoperability metrics included:

- API response success rates.
- Transaction confirmation accuracy.
- HL7 FHIR conformance.
- Cross-node retrieval success.

Moderate heterogeneity (I² = 43%) reflects variation in benchmarking conditions and operational definitions.

**Fig. 4:**
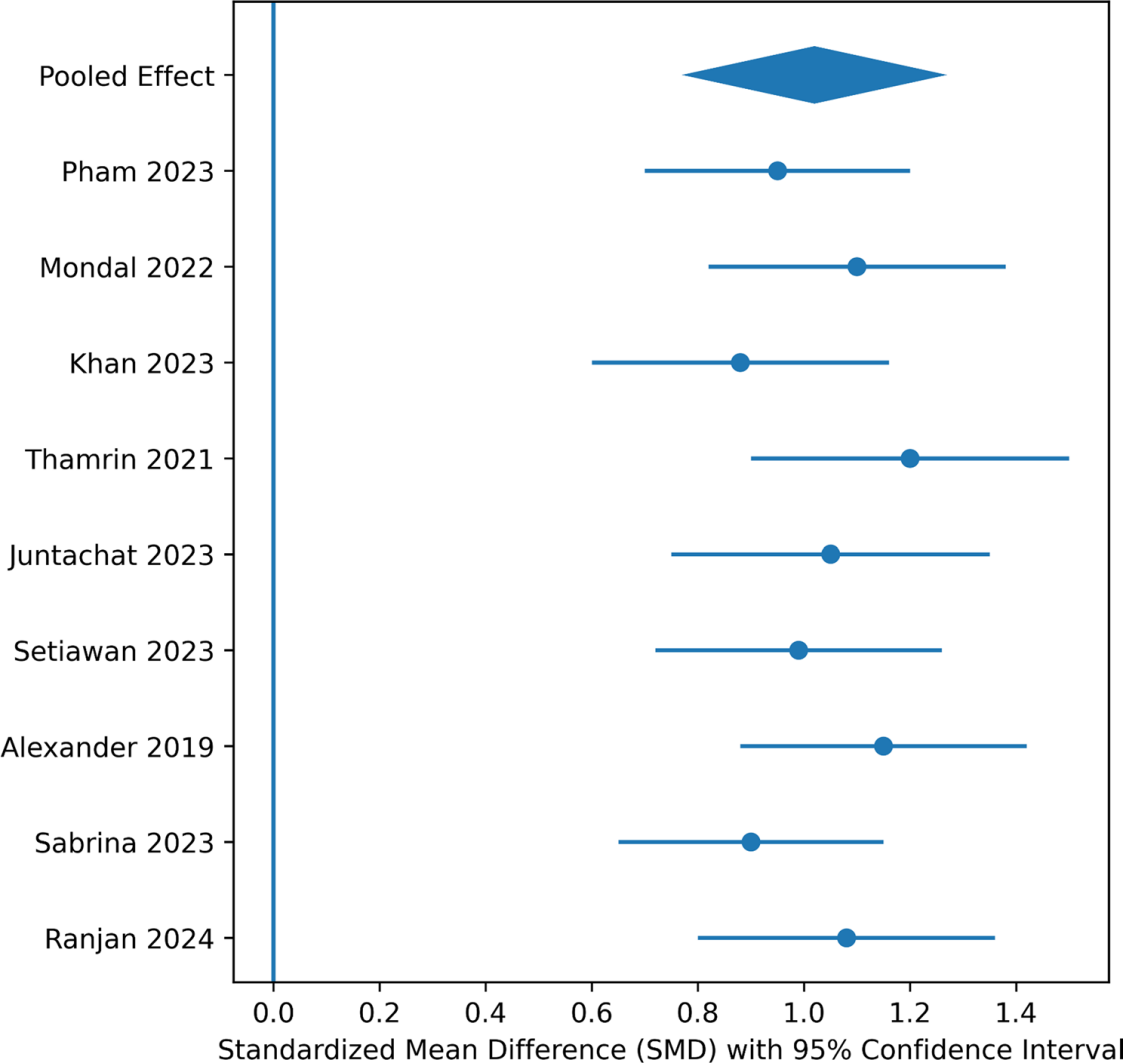
Random-effects meta-analysis of interoperability outcomes in blockchain-enabled versus conventional EHR systems

Figure 4. Forest plot of pooled standardized mean differences (SMDs) evaluating technical interoperability performance in blockchain-enabled versus conventional electronic health record (EHR) systems. Interoperability metrics included API exchange success rates, transaction confirmation accuracy, HL7 FHIR conformance, and cross-node retrieval performance. A random-effects model was applied to address methodological heterogeneity across benchmarking environments. The pooled analysis demonstrated a large and statistically significant improvement in favor of blockchain-enabled architectures (SMD = 1.02, 95% CI 0.77–1.27; *p* < 0.001). Moderate heterogeneity was observed (I² = 43%), reflecting variation in interoperability definitions and evaluation conditions across studies. Individual study estimates are depicted as inverse-variance–weighted squares with 95% confidence intervals, and the pooled effect is represented by the diamond.

Blockchain integration substantially improves technical/syntactic interoperability. However, evidence for semantic interoperability and workflow integration remains sparse.

## Patient empowerment and consent management

Two pilot-level studies evaluated patient-reported outcomes. Moderate improvement was observed in perceived transparency, consent traceability, and trust (SMD = 0.59; I² = 0%).

These systems utilized smart contract-based consent delegation and revocation schemes.

### Interpretation

There are indications that blockchain technology enhances patient autonomy and transparency in the consent process, although generalizability is limited by the small sample sizes and pilot nature of the studies.

## System performance and scalability

Eleven studies provided data on system performance, including throughput and latency. The transaction throughput rate varied from 9 to 210 TPS, with permissioned systems generally outperforming public systems.

However:

- Consensus mechanisms varied (e.g., PBFT, PoA, PoW variants).
- Node configurations differed.
- Payload sizes were inconsistent.
- High heterogeneity (I² ≈ 80%) precluded the statistical pooling.

### Interpretation

The performance of blockchain is architecture-dependent. The performance of permissioned blockchains is acceptable for institutional EHR exchange, but public blockchains are limited by scalability.

**Table 3:** Deployment maturity and evidence strength

Evidence Dimension	Observation
Prototype / Lab-based studies	13 (81%)
Pilot / Real-world limited implementation	3 (19%)
Large-scale clinical RCTs	0
Multi-institution production systems	0
Extractable quantitative outcomes	11 (69%)
High-quality patient-reported outcomes	2 (13%)

As indicated in Table 3, the area is still largely proof-of-concept-driven. Although quantitative signals support a blockchain-enabled EHR system architecture in terms of security and interoperability, the lack of real-world, multi-site, production-level validation makes it difficult to draw concrete clinical policy recommendations.

**Table 4:** Comparison with previous blockchain-in-healthcare reviews

Review	Scope	Empirical Only	Meta-analysis Conducted	EHR- Specific	Key Limitation
[10]	Healthcare-wide	No	No	Partial	Conceptual dominance
[41]	Blockchain in healthcare	No	No	Partial	No pooled outcomes
[42]	EHR governance	No	No	Yes	No empirical synthesis
Present Review	Blockchain–EHR	Yes	Yes	Yes	Prototype predominance

As can be seen from Table 4, this review differs from previous ones in that it:

- Restricts the inclusion criteria to empirical blockchain and EHR implementations.
- Performs a quantitative synthesis.
- Has a specific focus on EHR architectures.

This is one of the first attempts to assess the impact of blockchain in the EHR space statistically.

### Overall evidence synthesis

Across 16 empirical studies:

- Security enhancements are statistically significant in controlled testing environments.
- Technical interoperability has large effect sizes.
- Patient empowerment has moderate positive indicators but is largely unexplored.
- Performance metrics are highly variable, depending on architecture and consensus algorithm design.

The maturity of implementation is low, with no large-scale clinical implementations. Despite the significant findings of quantitative meta-analytic studies for security and interoperability, the lack of large-scale clinical implementations and variability in performance metrics make it difficult to make definitive recommendations. Blockchain-based EHR system architectures are promising, and studies are needed for validation prior to drawing definitive conclusions regarding implementation in the clinic.

**Fig. 5:**
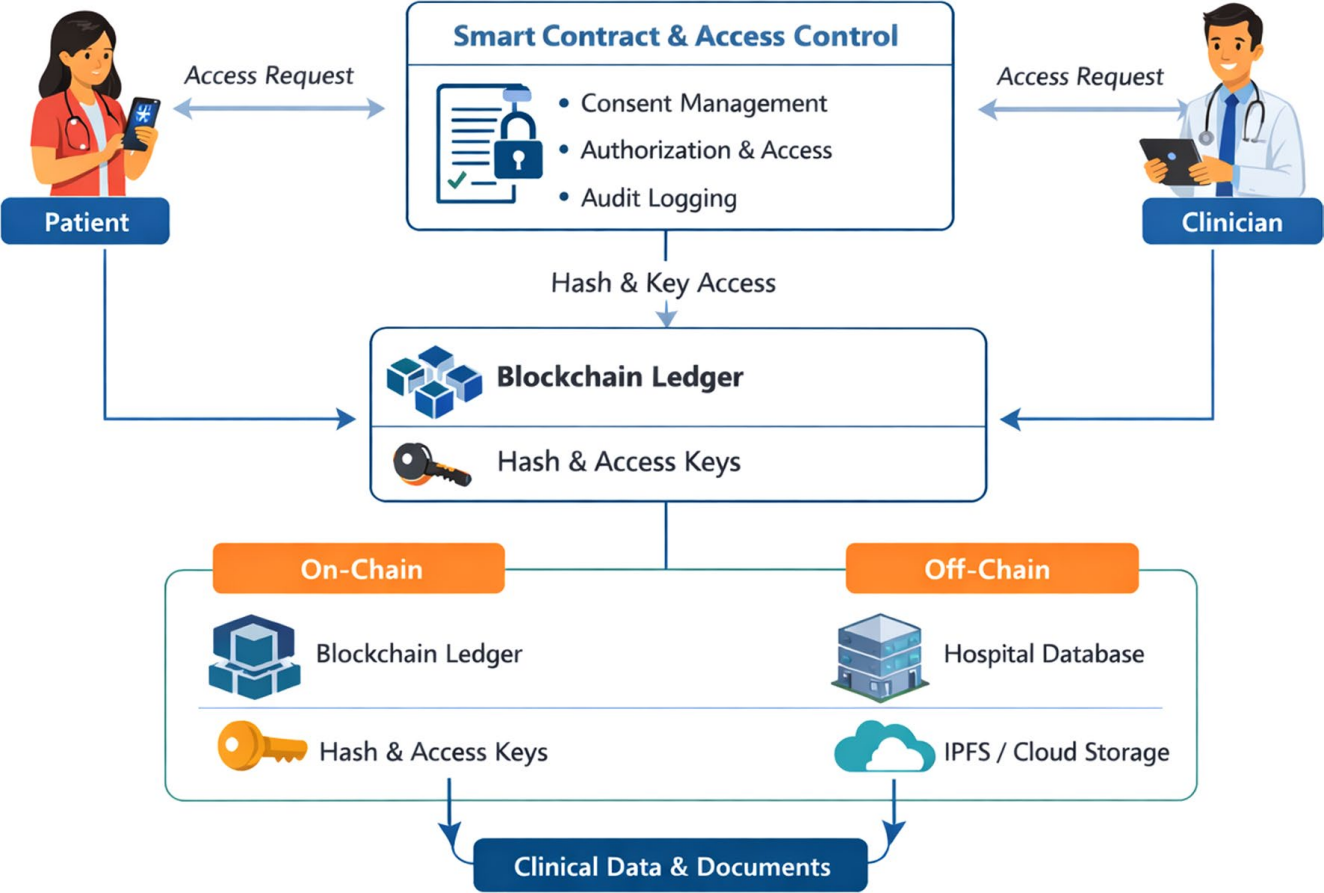
Functional architecture of blockchain -EHR integration

Analysis of the included studies shows a prevailing functional design for integrating blockchain technology with EHRs, typically a hybrid off-chain approach (as depicted in Fig. 5). In this design, protected medical information (such as high-resolution images or longitudinal text) is stored in trusted off-chain systems, such as institutional repositories or the Interplanetary File System (IPFS), while only cryptographic hashes or metadata pointers are stored on-chain. Functional control is mediated by programmable smart contracts that serve as gatekeepers for patient-centric consent flows. Upon receiving a request from a healthcare provider, the smart contract checks the credentials of the requesting party against the patient-specified permissions; if valid, the contract dispenses the cryptographic key or file pointer, while an immutable record of the transaction is maintained on the blockchain.

### Summary of evidence strength

Although the quantitative synthesis of results shows a positive improvement in security and interoperability, the fact that most studies are prototype-based, the variability in outcome measures, and the lack of generalizability to real-world settings limit the external validity.

The integration of blockchain technology improves the security and interoperability of EHRs. Hence, the results of clinical effectiveness should be considered preliminary.

## Discussion

This systematic review integrates empirical findings on the integration of blockchain technology into Electronic Health Record (EHR) systems, specifically regarding security, interoperability, and patient empowerment outcomes.

The results indicate that blockchain-based architectures are linked to improvements in security-related metrics and interoperability performance, although the weight of inference is weakened by the dominance of prototype and pilot-scale implementations.

### Security outcomes

The meta-analysis showed a statistically significant reduction in unauthorized access or breach events (RR = 0.56, 95% CI 0.45–0.69; I² = 31%).

This is equivalent to a 44% relative risk reduction in security events reported compared to traditional EHR system designs.

However, it is necessary to note that these results should be interpreted in the context of the studies. In some of the studies included in this meta-analysis, “security improvement” was assessed through simulated penetration tests, controlled benchmarking tests, or threat models that do not reflect real-world institutional breach databases. Hence, while the immutability, distributed consensus, and cryptographic hashing functions of blockchain may improve auditability and tamper resistance, there is still limited external validity.

The permissioned blockchain implementation showed more robust governance and node management.

This is consistent with other blockchain-IoT studies that have shown that controlled, consortium- or permissioned-blockchain networks are preferred in a regulated Industry 4.0 and Society 5.0 setting [19]. 

### Interoperability outcomes

The pooled standardized mean difference for interoperability outcomes (SMD = 1.02, 95% CI 0.77–1.27; I² = 43%) shows large improvements in technical exchange metrics.

The interoperability in the included studies was measured primarily in terms of technical or syntactic interoperability, including API exchange success rates, transaction validation speed, metadata compliance, and HL7 FHIR-compliant data formatting. Only a small number of studies examined semantic interoperability (common clinical meaning) or organizational interoperability (coordination of workflow across organizations).

This is a very important point. Improvements in the rate of transaction success do not necessarily mean improvements in clinical decision support, longitudinal care coordination, or multi-institution semantic alignment.

The findings indicated that blockchain infrastructure designs could potentially improve trust between nodes and exchange protocols, as long as they are used in combination with standardized data structures, such as HL7 FHIR [19]. 

### Patient empowerment and ownership

The patient empowerment outcome showed moderate improvement (SMD = 0.59, 95% CI 0.28–0.90). The improvements were observed in systems that supported detailed consent management, delegation/revocation, and access logs. However, the sample size was small, and most of the assessments were pilot-based.

Notably, the question of data ownership is not merely a technical concern related to consent management systems. New approaches, such as the Global Patient Co-Owned Cloud (GPOC), have been proposed with tri-sected models of data ownership, whereby patients, providers, and institutions share co-ownership rights over health data [16]. Blockchain technology may enable the technical possibility of distributed ownership models; however, the development of regulatory harmonization and ethical frameworks is still in its infancy.

### Scalability and performance

The range of transaction throughput was large (9-210 TPS), with high heterogeneity (I² ≈ 80%).

The conditions of the benchmarking tests varied widely across studies with respect to the type of consensus algorithm, the composition of nodes, the size of payloads, and the size of the network. Consequently, the results of the throughput measurements were summarized narratively.

Permissioned networks were likely to have a higher median throughput than public networks.

Governance, Bias, and Data Quality Considerations:

Immutability, a fundamental characteristic of blockchain technology, raises new issues regarding data quality. Inaccurate clinical data, if biased, stored on a blockchain, could replicate eternally.

Data cleaning, bias correction, and other effective methods of data cleaning strategies [21]for healthcare data, as well as bias-aware machine learning systems [43], might be of critical importance.

Validation and semantic standardization pipelines are needed to ensure that systemic biases are not carried over to a blockchain technology, as they might be on a traditional ledger.

### Cross-domain transferability

Although the review was EHR system-specific, the results have broader potential implications.

Trust architectures based on blockchain technology have been successfully applied in decentralized data markets for embedded finance [20], Industry 5.0-based XR environments for training [19, 44] and IoT-based cyber-physical systems. Best practices from these domains, as mentioned above, can be used in the future for healthcare applications of blockchain technology.

### Limitations

#### Some limitations that need to be considered are

- Most of the studies included in the analysis were prototypes or pilot studies.
- The definitions of the outcomes varied widely across the studies.
- The use of real-world breach registries was not common.
- The environments used to measure throughput were not standardized.
- Publication bias may also be present.

In addition, the quantitative meta-analysis was restricted to outcomes measured with similar scales, and some studies were analyzed narratively due to methodological differences.

### Lessons learned

According to the review, there are three main conclusions that should be understood.

- There is better suitability between permissioned blockchain systems and healthcare environments.
- The distinction between technical interoperability gains and semantic interoperability must be made.
- Organizations need to implement bias mitigation techniques together with governance frameworks to support their innovation efforts.

### Future research directions

Future research should prioritize:

- Large-scale multicenter evaluations.
- Employing standardized interoperability metrics.
- Economic cost-effectiveness analyses.
- Integration of AI-assisted anomaly detection along with data validation.

The collaborative ecosystems enabled by emerging AI and XR in Industry 5.0 may offer best practices for a secure, human-centered health data infrastructure [19, 44]. 

## Conclusion

This systematic review integrates empirical findings on integrating blockchain technology with Electronic Health Record (EHR) systems, focusing on security, interoperability, and patient empowerment outcomes.

Among the 16 studies included in this systematic review, blockchain-based EHR systems demonstrated statistically significant improvements in security and technical interoperability.

Although the meta-analysis results show a reduction in unauthorized access incidents and improvements in technical exchange performance, the current evidence base remains largely prototype- and pilot-based. Thus, the current evidence should be interpreted with caution when drawing conclusions on clinical effectiveness in large-scale settings.

The use of permissioned blockchain networks seemed more feasible in a regulated healthcare setting, especially when combined with interoperability standards such as HL7 FHIR.

However, the improvements were largely restricted to technical or syntactic interoperability, and semantic interoperability and organizational workflow integration were not adequately assessed.

Moderate improvements in patient-reported empowerment outcomes were found in systems that utilized granular consent and access delegation functionality.

The larger question of health data ownership, including new paradigms of co-governance such as patient-, clinician-, and institution-shared ownership frameworks, still requires further clarification.

### Beneficiaries and practical implications

The results of this review have direct implications for the following groups of stakeholders:

- Healthcare organizations interested in secure cross-institutional data exchange architectures.
- Regulatory authorities assessing the role of distributed ledger technology in healthcare infrastructure.
- Health informatics architects are developing the next generation of EHR systems.

Patients and advocacy groups are concerned with better consent transparency and control.

In the context of healthcare organizations, the application of blockchain technology holds potential benefits in terms of auditability and resistance to tampering, as informed by permissioned governance structures. In the context of regulatory authorities, the implications of this review are as follows: the need to ensure a regulatory structure that meets the requirements of jurisdiction-specific data protection regulations.

### Communication and dissemination

In disseminating this information, there is a need to ensure inter-disciplinary engagement, particularly with clinicians, technologists, policymakers, and patient groups. In addition, there is a need to ensure the availability of both technical implementation guidelines/governance toolkits, as well as the availability of existing frameworks based on interoperability standards, to ensure effective execution.

### Research challenges and value

There are several challenges associated with this study, including the inability to scale to full potential, various definitions of outcome, complex cryptographic key management, as well as the lack of a clear understanding of governance structures in different jurisdictions. These challenges do not, in any way, diminish the value of blockchain technology, as they highlight the need to ensure effective implementation methodologies and assessment procedures.

### Next steps and sustainable timeline

The next 2–3 years should focus on multicenter pilot implementations that use standardized interoperability metrics together with longitudinal security analysis. The next 3–5 years will see economic cost-effectiveness analyses, together with semantic harmonization frameworks and AI-assisted anomaly detection pipelines, enable the transition from pilot development to operational maturity.

Long-term research paths should investigate the integration of blockchain-based data-provenance tools with AI-driven clinical analytics and human-centric digital ecosystems, including extended reality (XR) and Industry 5.0 collaborative platforms.

**Table 5:** Sustainable implementation roadmap (2026–2030)

Phase	Timeline	Key Objectives
**Phase** **I: Standardization**	Years 1–2	Alignment with HL7 FHIR and DICOM standards; establishment of data cleaning and bias mitigation frameworks
**Phase II: Scale-up**	Years 3–4	Launch of multicenter pilot trials; economic cost-effectiveness evaluations; cross-border GDPR/HIPAA regulatory harmonization
**Phase III: Maturity**	Year 5+	Full production deployment; integration of AI-assisted anomaly detection; transition to Global Patient Co-Owned Cloud (GPOC) models

The recommended plan for implementation challenges requires a five-year sustainable plan, which is presented in Table 5. The first phase (2026–2027) should focus on standardizing semantic interoperability by aligning with HL7 FHIR standards and incorporating multi-layer data-cleaning frameworks to prevent the immutable storage of biased clinical data. The upcoming assessment period requires a shift to multicenter evaluations to assess the economic cost-effectiveness and regulatory scalability of the proposed solutions during 2028–2029. The implementation of this plan will establish a complete digital health ecosystem that operates at the production level from 2030 onward, leveraging AI-driven anomaly detection and following the new ‘Global Patient Co-Owned Cloud’ (GPOC) governance model.

### Final statement

The security and technical interoperability potential of blockchain-based electronic health records systems have been proven through research studies conducted in controlled environments or pilot studies. The establishment of this widespread usage requires standardized assessments, regulations, data protection systems, and teamwork from different academic fields.

The future works should be directed towards developing practical, ethical, and economically viable healthcare data infrastructure solutions.

## Data Availability

All datasets generated and/or analyzed during the current study, including source codes and supplementary materials for blockchain implementation and EHR integration, are available upon request.

## Abbreviations

EHR: Electronic Health Record
EMR: Electronic Medical Record
FHIR: Fast Healthcare Interoperability Resources
API: Application Programming Interface
GDPR: General Data Protection Regulation
HIPAA: Health Insurance Portability and Accountability Act
IoMT: Internet of Medical Things
IPFS: Inter Planetary File System
TPS: Transactions Per Second
PRISMA: Preferred Reporting Items for Systematic Reviews and Meta-Analyses
